# Quantification of Hepatitis E Virus ORF2 Protein by a Novel Sandwich ELISA

**DOI:** 10.3390/v16030393

**Published:** 2024-03-02

**Authors:** Sakthivel Subramaniam, Rafaelle Fares-Gusmao, David R. McGivern

**Affiliations:** Center for Biologics Evaluation and Research, US Food and Drug Administration, Silver Spring, MD 20993, USA; sakthive.subramaniam@fda.hhs.gov (S.S.); rafaelle.gusmao@fda.hhs.gov (R.F.-G.)

**Keywords:** hepatitis E virus, ORF2, biomarker, monoclonal antibodies, immunoassay, quantitative, sandwich ELISA, antigen detection, serology, biotin–streptavidin detection

## Abstract

Hepatitis E virus (HEV) causes acute hepatitis in humans, which can progress to chronicity in immunosuppressed individuals. Almost all reported HEV infections are caused by *Paslahepevirus balayani* genotypes 1–4. The structural ORF2 protein is the major antigen detected in the blood of HEV-infected individuals. ELISA assays to detect IgM antibodies to HEV are the first-line diagnostic tests; however, they showed variable performance with frequently discordant results. A qualitative HEV antigen (ORF2) ELISA is currently available for research use. Here, we report a novel quantitative sandwich ELISA to measure HEV ORF2 protein in 3 matrix types. An optimal pair of capture and detection antibodies was selected among 12 unique combinations tested. A sandwich ELISA protocol was developed using these mAbs and biotin–streptavidin technology. The protocol was further optimized to quantify ORF2 antigen in different matrices by interpolating from a standard curve with a linear range of 3.17 to 50.8 femtomoles/mL. Using this method, ORF2 protein was detected in the cell culture medium of Huh7 cells as early as 2–3 days after transfection with HEV genome RNA and in a medium of human hepatocytes infected with HEV. ORF2 antigen was readily detected in the first 2 weeks post-HEV infection in gerbil sera. In immunosuppressed gerbils, ORF2 was detected up to 6 weeks, and the levels were significantly higher between 3 and 6 weeks post-infection. HEV ORF2 antigen levels showed a strong positive correlation with HEV RNA levels in both cell culture medium and gerbil sera. Our novel sandwich ELISA detected at least 7.3 femtomoles/mL ORF2 protein in human plasma spiked with cell culture propagated HEV and detected ORF2 protein in human plasma samples that tested positive for HEV RNA but negative for anti-HEV antibodies. Further, the assay was nonreactive, with negative human plasma, and HBV or HCV-positive human plasma demonstrating specificity. Overall, our ORF2 antigen ELISA will be useful for quantifying ORF2 antigen in cell culture medium, gerbil serum, and human plasma. Further studies are warranted to evaluate its utility in HEV clinical diagnosis.

## 1. Introduction

Hepatitis E virus (HEV) is a major cause of acute hepatitis in humans worldwide [1]. Currently, there are eight distinct HEV genotypes (under genus *Paslahepevirus*) reported, but genotypes 1–4 (GT 1–4) were identified as the cause for the most human clinical cases [2,3]. GT1 and 2 are obligate human pathogens transmitted through contaminated drinking water, causing large-scale epidemic outbreaks of acute hepatitis in flood-prone regions of the world with poor sanitary infrastructure [4]. In contrast, GT 3 and 4 are zoonoses associated with sporadic cases of acute and chronic hepatitis E worldwide, which are mainly transmitted to humans through the consumption of undercooked organ meat (primarily pig products) and, rarely, through blood transfusions [5]. Despite a diverse host range, distinct epidemiological features, and genetic diversity, GT1–4 possess a single serotype [4]; therefore, well-developed serological assays can be useful for HEV diagnosis.

Anti-HEV IgM assays are used to diagnose acute hepatitis E in immunocompetent patients. However, the performance of these assays was found to be variable, and discordance between assays was noted [6,7]. Moreover, the IgM assays are not reliable for the diagnosis of chronic hepatitis E in immunosuppressed patients [8]. Therefore, the European Association for the Study of the Liver (EASL) recommends using a combination of anti-HEV IgM and HEV NAAT (nucleic acid amplification technology) to diagnose acute hepatitis E cases and the HEV NAAT only to diagnose chronic hepatitis E cases [8]. NAAT assays are also used for screening HEV infections in asymptomatic blood donors in European countries and Japan [9,10]. However, NAAT is not widely used in resource-limited countries for HEV diagnosis and screening because of the requirement for expensive specialized equipment and trained staff [11]. A qualitative HEV antigen (ORF2) serologic assay is commercially available for research use, and the diagnostic utility of this assay was investigated in few studies involving small cohorts of patients hospitalized with acute hepatitis E [12,13,14,15,16,17]. 

The qualitative ORF2 antigen assay detected acute hepatitis E cases with a sensitivity of 91–100% [13,15]. The assay was able to discriminate acute and chronic hepatitis E cases of GT3 infection with a sensitivity of 95% and specificity of 88% [16]. The ORF2 antigen assay also discriminated self-limiting GT1 infections from fulminant hepatic failure progression with 100% sensitivity and 89% specificity [17]. The positive and negative predictive values of the ORF2 antigen ELISA for the clinical diagnosis of acute GT1 outbreaks were found to be 60–81% and 88–100%, respectively [12]. HEV ORF2 antigen may be a useful biomarker of active HEV infection in cases where prolonged IgM response is observed after the resolution of acute hepatitis E [14]. A logistic regression model integrating three serological markers of HEV infection, ORF2 antigen, anti-HEV IgM, and ALT, improved the sensitivity (89.5%) and specificity (95.2%) of the clinical diagnosis of ongoing HEV infections compared with an anti-HEV IgM assay or HEV antigen assay [18]. However, the utility of ORF2 antigen in hepatitis E diagnosis is yet to be determined by developing new, improved assays and conducting studies involving large patient cohorts.

The HEV ORF2 protein was detected at high levels in the sera of human patients as well as rhesus macaques experimentally infected with HEV [19,20]. Three distinct ORF2 isoforms were identified in the sera of HEV-infected persons: (1) secreted glycosylated ORF2 (ORF2g), (2) a cleaved ORF2 (ORF2c), and (3) the capsid-associated ORF2 (ORF2i) [19,20,21]. The former two isoforms are the major antigens in blood and primary targets of HEV antigen ELISA [19]. In this study, we developed and optimized a quantitative sandwich ELISA protocol using two novel monoclonal antibodies specific to HEV ORF2 antigen and the biotin–streptavidin technology to improve the sensitivity of detection. The assay was tested for quantifying ORF2 antigen in a cell culture medium of human hepatocytes, serum from HEV-infected gerbils, and ORF2-spiked human plasma.

## 2. Materials and Methods

### 2.1. Human Plasma Specimens

Whole Blood was collected from volunteer donors in citrate phosphate dextrose at the NIH Division of Transfusion Medicine under approval from the NIH and FDA Institutional Review Boards (IRBs). Plasma was prepared from Whole Blood and immediately stored at −80 °C until use.

Plasma derived from deidentified blood donor samples reactive to markers of HBV or HCV infection was purchased from the American Red Cross Infectious Disease Repository (Gaithersburg, MD, USA). Plasma specimens reactive to HEV were purchased from SeraCare/LGC Diagnostics, Milford, MA, USA (AccuSet HEV Performance Panel, 0820-0503/Batch #10640609). The plasma samples were aliquoted and stored at −80 °C until use. Frozen samples were thawed and diluted directly in blocking buffer for sandwich ELISA analysis.

### 2.2. Animal Studies

The animal studies were conducted in strict adherence to the recommendations in the Guide for the Care and Use of Laboratory Animals of the National Institutes of Health. The animal study protocols were reviewed and approved by FDA White Oak Animal Care and Use Committee (ACUC), Silver Spring, USA, protocol numbers 2019-12 and 2020-02.

### 2.3. Preparation of Recombinant ORF2 Antigens

The DNA sequence encoding ORF2 amino acids 422–637 (US-2 strain, GenBank Accession No: AAD15816.1, NCBI (Bethesda, MD, USA)) was codon optimized and chemically synthesized (IDT technologies, Coralville, IA, USA). The ORF2 DNA was inserted into the pET28a+ expression vector (Novagen, EMD Millipore, Burlington, MA, USA). The resulting plasmid was used to transform BL21 (DE3) *E. coli*. ORF2 expression was induced by IPTG induction. Cells were lysed, and ORF2 was purified from lysates by His tag affinity chromatography using HisPur Ni-NTA superflow agarose resin (Thermofisher Scientific, Waltham, MA, USA). The purified ORF2 protein was desalted and exchanged with PBS by ultrafiltration or dialysis. The protein concentration was measured by Bradford assay, and the protein was aliquoted for single use and stored at −80 °C.

### 2.4. Preparation of Mouse Monoclonal Antibodies against the ORF2 Antigen

Six-week-old female Balb/c mice were immunized with 50 µg ORF2 antigen emulsified with Titermax gold adjuvant at 1:1. Mice received booster immunizations at 4 and 6 weeks with 25 µg antigen emulsified with Freund’s incomplete adjuvant at 1:1. Antibody responses in mice were assessed by an indirect ELISA at 1 week after each booster immunization. At 10 weeks, mice were injected intravenously with 5–10 µg antigen. Three days later, mice were euthanized, and spleens were harvested for splenocyte preparation. Splenocytes were fused with Sp2/0-Ag14 myeloma cells (ATCC) to generate hybridomas. A panel of 1000 hybridoma clones was generated using ClonaCell-HY Hybridoma kit (Stem Cell technologies, Cambridge, MA, USA). Hybridoma culture supernatants were screened by an indirect ELISA for ORF2 antibodies. Five hybridoma clones positive for ORF2 antibodies were identified, cultured in bulk, and antibodies were purified from the culture supernatants using PureProteome Protein G magnetic beads (Millipore sigma, Burlington, MA, USA) according to the manufacturer’s instructions. Only four ORF2 antibodies were tested for antigen capture and detection. The antibodies for antigen capture were stored at 4 °C in Tris-glycine buffer containing 0.02% sodium azide. The antibodies used in detection were desalted, buffer exchanged with PBS, and conjugated with biotin using Lightning-Link Biotinylation Kit (Type A) (Abcam, Waltham, MA, USA) according to the manufacturer’s instructions. The biotin-conjugated antibodies for detection were stored at 4 °C in PBS mixed with sodium azide at 0.02% final concentration.

### 2.5. Sandwich ELISA Protocol Development

A 96-well microplate (Nunc Maxisorp, Thermofisher Scientific, Waltham, MA, USA) was coated with capture antibody (200 ng/100 μL/well, diluted in ELISA coating buffer (Bio-Rad, Hercules, CA, USA)) overnight at RT. After 3 washings with PBS-Tween 20 (0.05%) (PBS-T), blocking buffer (5% non-fat milk, 150 μL/well) was added and incubated for 1 h at 37 °C. The blocking buffer was decanted, and serially diluted recombinant ORF2 p216 antigen (0.1, 1, 10, 100, and 1000 ng/mL; 100 μL per well) was added and incubated overnight at 4 °C. Duplicate wells were prepared for each dilution. After 3 washings with PBS-T, biotin-conjugated detection antibody (1 μg/mL, 100 μL/well) was added and incubated for 1 h at 37 °C. After 5 washings with PBS-T, Streptavidin–HRP (1:20,000 dilution, 100 μL/well, Thermofisher Scientific, Waltham, MA, USA) was added and incubated for 1 h at 37 °C. After 5 washes with PBS-T, SureBlue TMB microwell peroxidase substrate (100 μL/well, KPL, SeraCare/LGC Diagnostics, Milford, MA, USA) was added and incubated for 5–30 min at RT in the dark. The reaction was stopped with 1N HCl (100 μL/well). Absorbance was measured at 450 nm in a microplate reader (Biotek Synergy neo2, Agilent, Santa Clara, CA, USA).

### 2.6. Screening of Matched Antibody Pairs

The 4 capture antibodies (c7A10, c7D3, c8A8, c9D4) were paired with 4 detection antibodies (d7A10, d7D3, d8A8, d9D4) and screened in the sandwich ELISA following the protocol indicated above. Serially diluted recombinant ORF2 p216 antigen (0.1, 1, 10, 100, and 1000 ng/mL; 100 μL per well) and blocking buffer without antigen served as positive samples and negative control in the assay, respectively. The antibody pair showing largest ratios of OD450 values between the positive samples and negative control was selected as the optimal matched antibody pair for the sandwich ELISA.

### 2.7. Sandwich ELISA Protocol Optimization

The optimal antibody concentrations for both capture and detection antibodies were determined by checkerboard titration. Different concentrations of the capture antibody (2, 4, 6, 8, 10 µg/mL) and detection antibody (0.5, 1, 2 µg/mL) were tested in the sandwich ELISA. Serially diluted recombinant ORF2 p216 antigen (0.1, 1, 10, 100, and 1000 ng/mL; 100 μL per well) and blocking buffer without antigen served as positive samples and negative control in the assay, respectively. The antibody concentrations showing the largest difference in OD450 values between the positive samples and negative control were selected. Similarly, the different concentrations of Streptavidin–HRP (1:4000, 1:10,000, 1:20,000) were tested in the sandwich ELISA, and the concentration showing the largest difference in OD450 values between positive samples and negative control was selected.

### 2.8. Generation of Standard Curves

The optimized sandwich ELISA protocol was followed to generate standard curves for ORF2 quantification. Recombinant US-2 ORF2 antigen (prepared in-house) was serially diluted 2-fold with blocking buffer (10, 5, 2.5, 1.25, 0.625, 0.312, 0.156, 0 ng/mL equivalent to 203.2, 101.6, 50.8, 25.4, 12.7, 6.35, 3.17, and 0 fmoles/mL, respectively). The standard curves were calculated with ORF2 antigen concentration on the *X*-axis and OD450 values on the *Y*-axis. The ORF2 antigen was quantified by interpolating from standard curve and expressed as fmoles/mL. The limit of detection (LOD) of ORF2 antigen in the ELISA was calculated using 34 negative serum samples obtained from 18 gerbils or 10 negative plasma samples obtained from 10 blood donors by the following formula: Mean Optical Density (OD) or Mean quantity (fmoles/mL) + (3× Standard Deviation (SD)). For gerbil serum samples, the calculated LOD is 6.2 fmoles/mL. For human plasma samples, the calculated LOD is 6.3 fmoles/mL or 0.011 (OD).

### 2.9. Cross-Reactivity Analysis

The cross-reactivity of the capture and detection antibodies to the ORF2 antigens of *Paslahepevirus balayani* (formerly HEV-A) GT1 (Genbank: AF444002), GT3 (Genbank: AF060669), GT4 (Genbank: HQ634346), and *Rocahepevirus ratti* (formerly HEV-C1 or rat HEV; Genbank: MK050105) was measured by the optimized sandwich ELISA. The ORF2 antigens were expressed in bacteria as described above. Two-fold serially diluted recombinant ORF2 p216 antigen (80–0.078 ng/mL; 100 μL per well) were tested, and blocking buffer without antigen served as negative control in the assay. The ratios of OD450 values between positive samples and negative control were calculated for different ORF2 antigens.

### 2.10. HEV RNA Preparation and Transfection in Huh7 Cells

In vitro, transcribed HEV Kernow C1 p6 RNAs or p6 GND (replication-incompetent) RNAs were prepared and electroporated into Huh7 S10-3 cells, as previously described [22]. Capped and uncapped HEV Kernow C1 p6 RNAs were prepared with or without ARCA (Anti-Reverse Cap Analog, Thermofisher Scientific, Waltham, MA, USA), respectively. Capped p6 GND RNAs were prepared with ARCA to serve as negative control. The Huh7 culture medium was collected and replaced with an equivalent volume of fresh medium every 2 days. The harvested culture medium was centrifuged at 500× *g* for 5 min to remove cell debris and stored at −80 °C until analysis. At the time of sandwich ELISA analysis, Huh7 culture supernatant was thawed and diluted up to 1:400 in the blocking buffer. Culture supernatant from capped p6 GND RNA transfected cells (at all time points) and from capped p6 RNA transfected cells (at 0 days post-transfection) were confirmed negative for ORF2 antigen at the lowest dilution 1:5 in the sandwich ELISA.

### 2.11. HEV Infection in Human Hepatocytes

Human hepatocytes (HH) isolated from chimeric mice with humanized livers were purchased from Phoenix Bio (Edmonton, AB, Canada), cultured on 24 well plates, and maintained according to the manufacturer’s instructions. HH cultures were infected with the genotype 3 HEV Kernow C1 p6 (Genbank JQ679013) at a MOI (multiplicity of infection) of 0.006. The MOI is calculated using focus forming units (FFU)/mL of the virus stock. The cell culture medium was collected and replaced with fresh medium every 1–4 days. The culture medium was subjected to centrifugation at 500× *g* for 5 min to remove cell debris and stored at −80 °C until analysis. HH culture supernatant was diluted up to 1:25 in the blocking buffer for sandwich ELISA analysis. Mock-infected HH culture supernatants and HEV-infected HH culture supernatants at 0 days post-infection were confirmed negative for ORF2 antigen at the lowest dilution of 1:5 in the sandwich ELISA.

### 2.12. HEV RT-qPCR

RNA was isolated from serum and cell culture supernatant using the QiaAmp Viral RNA mini kit (Qiagen) according to the manufacturer’s instructions. Frozen samples were thawed and adjusted to 140 µL with RNase-free water before RNA isolation. RNA was used fresh in RT-qPCR reactions or stored at −80 °C until use. Reverse Transcription, Quantitative Polymerase Chain Reaction (RT-qPCR) for HEV was based upon a previously published method [23] with modifications to increase sensitivity [24]. A standard curve was generated using a HEV RNA standard (full-length Kernow C1 p6; Genbank JQ679013) serially diluted 10-fold with nuclease-free water. “No template control” (NTC) was used as negative control and HEV RNA standard was used as positive control in each RT-qPCR plate. HEV RNA copies were calculated by interpolating from the standard curve and expressed as copies per mL.

### 2.13. Quantification of ORF2 Antigen in Gerbil Serum Samples

Clotted gerbil blood specimens were collected during a previously reported animal experiment [25]. Serum samples were prepared by centrifugation at 1500× *g* for 10 min at RT. Aliquots of serum samples were prepared for single use and stored at −80 °C. Serum samples were diluted up to 1:6400 in the blocking buffer for sandwich ELISA analysis. The serum samples from negative control gerbils were confirmed negative for ORF2 antigen at the lowest dilution of 1:10 in the sandwich ELISA.

### 2.14. Preparation of ORF2 Antigen-Spiked Human Plasma Samples

Clotted blood samples were obtained from 4 different human donors through NIH blood bank. Fresh plasma was prepared by centrifugation at 1500× *g* for 10 min at 4 °C. Plasma was spiked with culture medium from HEV-infected human hepatocyte cultures containing ORF2 antigen (414–608 fmoles/mL) at different volume ratios (1:2, 1:4, 1:8, 1:16, 1:32, 1:64). The spiked plasma was mixed thoroughly, aliquoted for single use and stored at −80 °C until further use. Spiked plasma samples were thawed and diluted 1:10 in the blocking buffer for sandwich ELISA analysis. Non-spiked plasma samples served as negative controls in the assay.

## 3. Results

### 3.1. Selection of Monoclonal Antibodies for HEV ORF2 Antigen Sandwich ELISA

**Figure 1:**
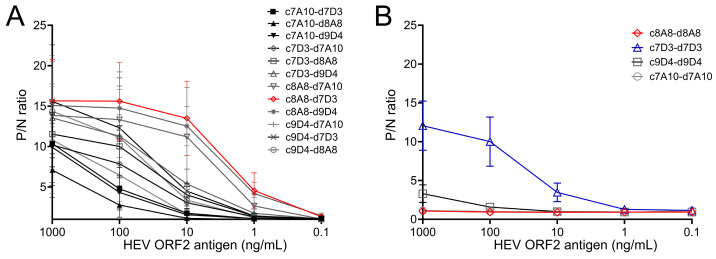
Sandwich ELISA development. (**A**) Identification of optimal capture antibody and detection antibody pair among 12 unique combinations tested in sandwich ELISA. The optimal capture and detection antibody pair is indicated as red symbols with a connecting line. (**B**) Evaluation of blocking of detection antibody binding to ORF2 p216 antigen by prior capture antibody binding to the antigen. Four same clone combinations of capture and detection antibody were tested. In both unique and same antibody combinations tested, the capture antibody is unconjugated, and the detection antibody is biotinylated to facilitate detection by Streptavidin–HRP system. ‘c’ and ‘d’ prefixes were added before antibody clone names to distinguish the coating and detection antibody clones, respectively. Each data set (indicated with a different symbol) is derived from testing one capture and detection antibody pair in the sandwich ELISA format using 10-fold serially diluted recombinant ORF2 p216 antigen. P/N ratio indicates the ratio of the absorbance (OD450) of the positive sample to that of negative control (blocking buffer). Higher P/N ratios indicate higher levels of nonoverlapping binding of capture and detection antibodies to the recombinant ORF2 p216 antigen in the sandwich format. Data are from at least 3 independent experiments, and ELISA was performed with 2 technical replicates per sample. Error bars represent standard deviation.

The experimental design of conducted experiments is illustrated in Supplementary Figure S1. We established a total of five mouse hybridoma clones that produced monoclonal antibodies (mAbs) specific to HEV ORF2 p216 antigen. Among them, four mAbs (7A10, 7D3, 8A8, 9D4) were found to be reactive to HEV ORF2 p216 antigens of both GT1 and GT3 and were selected for further characterization in a sandwich ELISA. The sandwich ELISA requires a pair of antibodies (for capture and detection) binding different non-overlapping epitopes of ORF2 antigen, and both antibodies should have a high affinity to the antigen. In the biotin–streptavidin technology, the biotinylation of the detection antibody should not affect the antibody binding affinity to the antigen. Therefore, we evaluated 16 combinations (4 capture × 4 detection mAbs) in competition experiments in the sandwich ELISA. Out of 12 unique antibody (Ab) combinations tested (Figure 1A), the most effective pair of antibodies detecting ORF2 antigen was 8A8 capture mAb and 7D3 detection mAb (Figure 1A). Among the four Ab combinations where the same mAb was used as capture and detection Abs, 8A8 and 7A10 mAbs did not detect the ORF2 antigen, while 7D3 and 9D4 mAbs detected the ORF2 antigen weakly in the sandwich ELISA (Figure 1B).

### 3.2. Optimization of Sandwich ELISA to Increase the Sensitivity of ORF2 Antigen Detection

**Figure 2:**
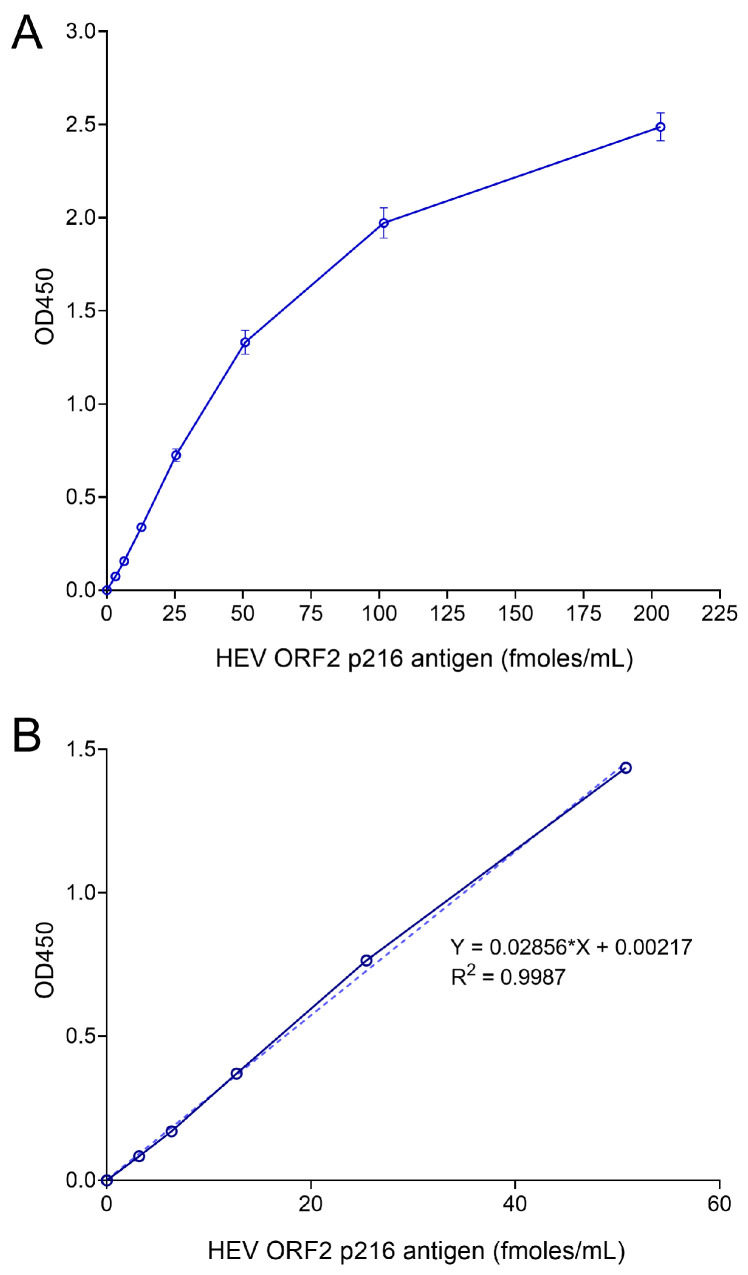
Sandwich ELISA optimization for quantification of HEV ORF2 antigen. (**A**) Standard curve generation with optimized sandwich ELISA protocol using 2-fold serially diluted recombinant ORF2 p216 antigen. Data are representative of at least 3 independent experiments. Error bars indicate standard error of mean. (**B**) Determination of the linear range of ORF2 protein quantification using the standard curve. A representative linear standard curve (dotted line) was shown, calculated using simple linear regression. The standard curve equation is in form of y = mx ± b (m is the slope and b is the y-intercept), and the goodness of standard curve fit was measured with the coefficient of determination (R^2^), which is predetermined to be at least 0.99. Due to presence of different ORF2 isoforms and possible degradation products in the biological samples, a common unit of femtomoles (of ORF2 antigen)/milliliter (fmoles/mL) was used to quantify ORF2 antigen.

Checkerboard titration analysis showed that the optimal concentrations of capture 8A8 and detection 7D3 mAbs were found to be 10 µg/mL and 2 µg/mL, respectively (Supplementary Figure S2). Additional experiments showed the optimal Streptavidin–HRP concentration (1:4000), incubation temperature, and duration of antibody coating (overnight at RT) and antigen capture (overnight at 4 °C), which, overall, improved the sensitivity of the sandwich ELISA, consistently detecting recombinant the ORF2 p216 antigen at least 3.17 fmoles/mL (Figure 2A). A standard curve was generated plotting the ORF2 antigen concentrations (fmoles/mL) on the horizontal axis and OD450 values on the vertical axis. The linear range of the standard curve ranged from 3.17 fmoles/mL to 50.8 fmoles/mL (R^2^ > 0.99) (Figure 2B).

### 3.3. Sensitivity and Specificity of the Sandwich ELISA for ORF2 Antigen of Different HEV Genotypes

**Figure 3:**
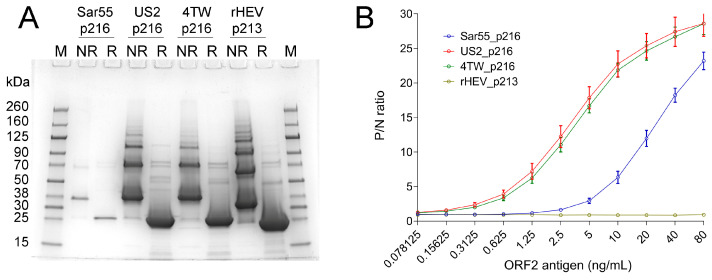
Detection of ORF2 antigen of major HEV genotypes in optimized sandwich ELISA (**A**) SDS-PAGE and Coomassie blue staining analysis of purified recombinant ORF2 antigens of HEV-A genotypes 1, 3, 4, and HEV-C1 (rat HEV). NR—Non-reduced, not-boiled; R—Reduced and boiled. The indicated molecular sizes were obtained with pre-stained protein ladder relevant only to completely denatured proteins. (**B**) Determination of cross-reactivity of the optimized ORF2 sandwich ELISA using 2-fold serially diluted recombinant ORF2 antigens of HEV-A genotypes 1, 3, 4, and HEV-C1 (rat HEV). The nomenclature of each data set includes strain name and molecular size of the corresponding ORF2 subunit antigen following the prefix ‘p’ for protein. P/N ratio indicates the ratio of the absorbance (OD450) of the positive sample to that of negative control (blocking buffer). The data represent mean ± standard deviation obtained from 3 independent experiments.

The sandwich ELISA was further evaluated for cross-reactivity and sensitivity in detecting ORF2 antigens of GT1, GT3, GT4, and rat HEV-C1. The ORF2 p213-p216 proteins were prepared in bacteria and purified, and their molecular size was confirmed by SDS-PAGE analysis (Figure 3A). The ORF2 antigen from GT3 and GT4 was detected at concentrations as low as 0.156 ng/mL, while the ORF2 antigen from GT1 was detected as low as 2.5 ng/mL (Figure 3B). The sandwich ELISA did not detect ORF2 antigen from rat HEV-C1 (Figure 3B). Overall, the sandwich ELISA is specific to ORF2 antigens from the major HEV genotypes (GT1, GT3, and GT4), showing higher sensitivity for GT3 and GT4.

### 3.4. Quantification of ORF2 Antigen in HEV-Infected Hepatocyte Culture Medium

**Figure 4:**
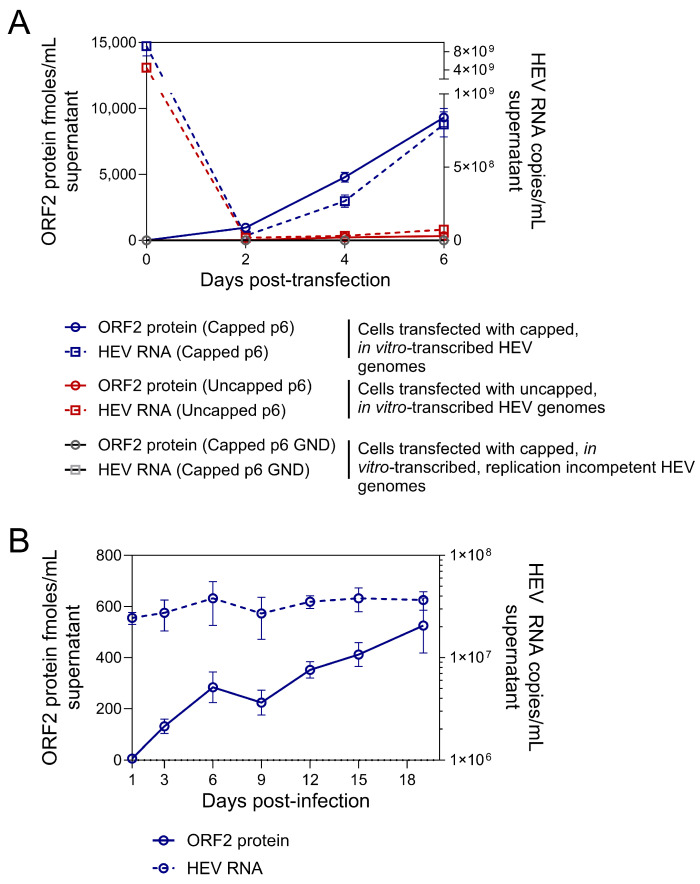
HEV ORF2 protein quantification in culture supernatants of HEV-infected hepatocytes. (**A**) ORF2 protein concentrations in the culture supernatants of Huh-7 cells transfected with full-length HEV RNAs (either capped or uncapped p6) or replication-incompetent HEV RNAs (capped p6 GND). HEV RNA concentrations were determined by RT-qPCR. Both ORF2 protein and HEV RNA were measured from same sample every 2 days post-HEV RNA transfection. Blue and red symbols represent data from capped or uncapped HEV RNA transfections, respectively. Grey symbols represent data from capped, replication-incompetent HEV RNAs. The data are mean ± standard error obtained from at least 3 independent experiments. RT-qPCR and ELISA were performed with 2 technical replicates per sample. (**B**) ORF2 protein concentrations in the culture supernatants of Primary Human Hepatocyte (PHH) cells infected with purified HEV particles. HEV RNA concentrations were determined by RT-qPCR. Both ORF2 protein and HEV RNA were measured from same sample every 2–4 days post-HEV infection. The data are mean ± standard error obtained from 2 independent experiments. RT-qPCR and ELISA were performed with 2 technical replicates per sample. In both figure panels, the solid line indicates HEV ORF2 protein data, and the dotted line indicates HEV RNA data. Left y-axis indicates ORF2 protein concentration expressed as femtomoles (fmoles) per mL of culture supernatant, and right y-axis indicates HEV RNA copies per mL of culture supernatant.

The ORF2 antigen was measured by sandwich ELISA in the culture medium of Huh7 cells transfected with in vitro transcribed HEV RNA (Figure 4A). At 2 days post-transfection (dpt), the ORF2 protein was detected (with a mean of 742 fmoles/mL) in the culture medium from cells transfected with capped HEV genome RNA but not in medium from cells transfected with replication-incompetent HEV genomic RNA carrying a mutation (GND) in the polymerase active site (Figure 4A). The ORF2 protein levels reached a mean of 10,408 fmoles/mL at 6 dpt in the culture supernatants from cells transfected with capped HEV RNA compared to a mean of 306 fmoles/mL in the culture supernatants from cells transfected with uncapped HEV RNA (Figure 4A). The culture supernatants from cells transfected with replication-incompetent HEV genomic RNA remained negative for ORF2 protein until 6 dpt (Figure 4A). The ORF2 protein levels strongly correlated with HEV RNA levels in the Huh7 culture supernatants (Pearson’s r = 0.89, *p* < 0.0001, 95% CI = 0.77–0.95), and the ratio of ORF2 protein (fmoles)/HEV RNA (million copies) in culture supernatants ranged between 7 and 59.9. ORF2 protein was also detected in the culture medium of human hepatocyte (HH) cultures infected with HEV GT3, albeit at lower levels as compared with Huh7 cells transfected with HEV RNA (Figure 4B). From 3 days post-infection (3 dpi), the ORF2 protein was detected in the HH culture medium, reaching a peak at 19 dpi (419 to 487 fmoles/mL) (Figure 4B). Consistent with the results obtained from Huh7 cells, the ORF2 protein levels correlated with HEV RNA levels in the HH culture medium, and the ratio of ORF2 protein (fmoles) to HEV RNA (million copies) ranged between 5.6 and 32.2. With these results, we can conclude that the sandwich ELISA detected full-length mature ORF2 protein secreted into the culture medium of HEV-infected human hepatocytes in vitro.

### 3.5. Quantification of ORF2 Antigen in Sera of Gerbils Infected with HEV

**Figure 5:**
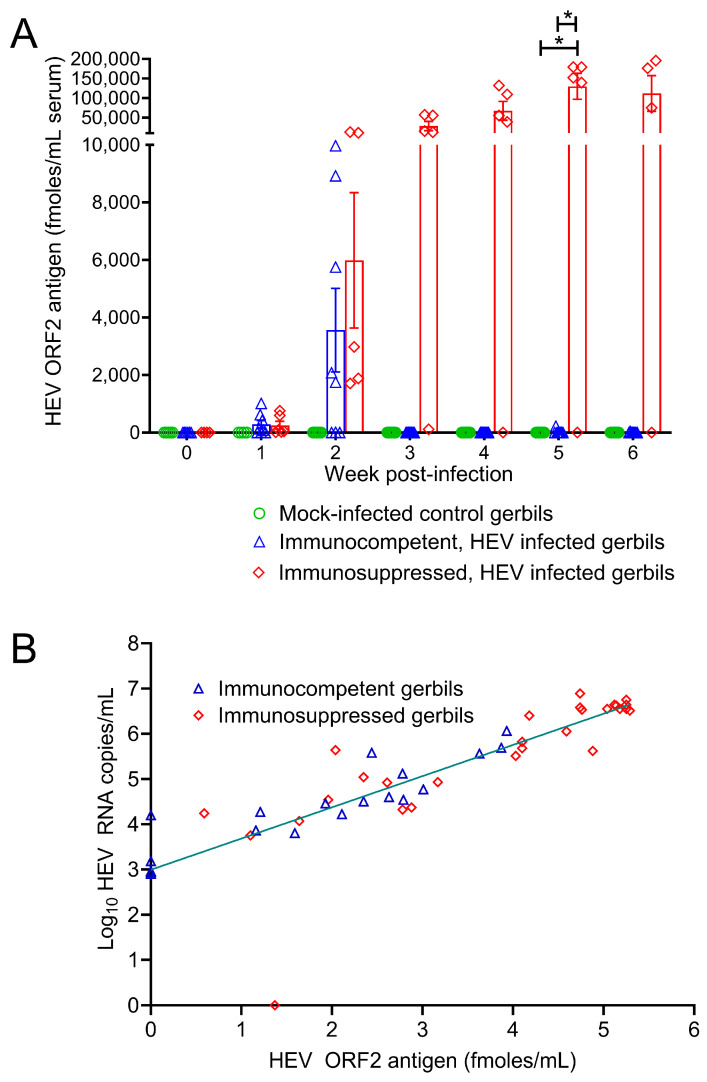
HEV ORF2 protein quantification in sera of immunocompetent and immunosuppressed gerbils infected with HEV. (**A**) The ORF2 protein quantities in gerbil sera were measured at weekly time points post-HEV infection. To assess the influence of adaptive immune responses on HEV ORF2 protein levels, general immunosuppression was induced in gerbils by controlled release tacrolimus pellet implanted at a subcutaneous neck site 2 weeks prior to HEV infection. Y-axis indicates HEV ORF2 protein expressed as femtomoles (fmoles)/milliliter of serum samples. Data represent mean ± standard error of mean calculated from 5 to 8 gerbils per treatment group. The statistical significance was tested with mixed effects model along with Greenhouse–Geisser correction and Tukey’s multiple comparison test. Asterisk indicates statistical significance at *p* < 0.05. (**B**) Simple linear regression analysis of ORF2 protein levels versus HEV RNA levels in sera of immunocompetent and immunosuppressed gerbils infected with HEV. Each data point represents both HEV ORF2 antigen (femtomoles (fmoles)) and HEV RNA (copies) per milliliter of same serum sample. Blue triangles indicate data from immunocompetent gerbils and red diamonds indicate data from immunosuppressed gerbils. The teal line is the regression line fitted with all data points from both immunocompetent and immunosuppressed gerbils.

The ORF2 antigen was measured in serum samples of gerbils infected with HEV GT3 (Kernow C1 strain [fecal derived], 1 × 10^7^ genome equivalents/animal intraperitoneally) with or without induced immunosuppression. The assay LOD for the sandwich ELISA was calculated with negative gerbil serum samples (*n* = 34; serial samples collected from 18 different gerbils) and found to be 6.2 fmoles/mL (Mean ± 3 SD). In the immunocompetent gerbils (*n* = 8), the ORF2 protein levels reached their highest at 2 weeks post-infection (wpi), ranging from 1754 to 9968 fmoles/mL serum (Figure 5A). In the immunosuppressed gerbils (*n* = 5), similar levels of ORF2 protein were detected at 2 wpi; however, the protein levels continued to increase beyond 2 wpi and reached a peak value of 139,522 to 195,854 fmoles/mL at 5–6 wpi (Figure 5A). In both immunocompetent and immunosuppressed gerbils, anti-HEV ORF2 antibodies were absent in sera until 2 wpi [25]. However, anti-HEV ORF2 antibodies (both IgM and IgG) were detected in the serum of most immunocompetent gerbils from 3 wpi onwards, but they were absent or at barely detectable levels in the serum of most immunosuppressed gerbils [25]. A Pearson correlation analysis showed a strong positive correlation between ORF2 antigen and HEV RNA levels in immunocompetent gerbil sera (Pearson’s r = 0.90, 95% CI = 0.75–0.96, *p* < 0.0001). However, only a moderate positive correlation was found between ORF2 antigen and HEV RNA levels in immunosuppressed gerbil sera (Pearson’s r = 0.74, 95% CI = 0.51–0.87, *p* < 0.0001). Using a simple linear regression analysis, the HEV RNA (copies/mL) can be predicted from HEV ORF2 (fmoles/mL) in immunocompetent gerbil sera with a reasonable accuracy using the equation Y = 99.73X + 22343 (R^2^ = 0.80) (Figure 5B). However, the HEV RNA (copies/mL) cannot be reliably predicted from HEV ORF2 (fmoles/mL) in immunosuppressed gerbil sera (R^2^ = 0.55) (Figure 5B). Overall, the ORF2 protein was detected at high levels in the serum of gerbils infected with HEV GT3, and the protein levels were strongly correlated with HEV RNA levels in the serum of immunocompetent gerbils.

### 3.6. Quantification of HEV ORF2 Antigen in Human Plasma Samples

**Figure 6:**
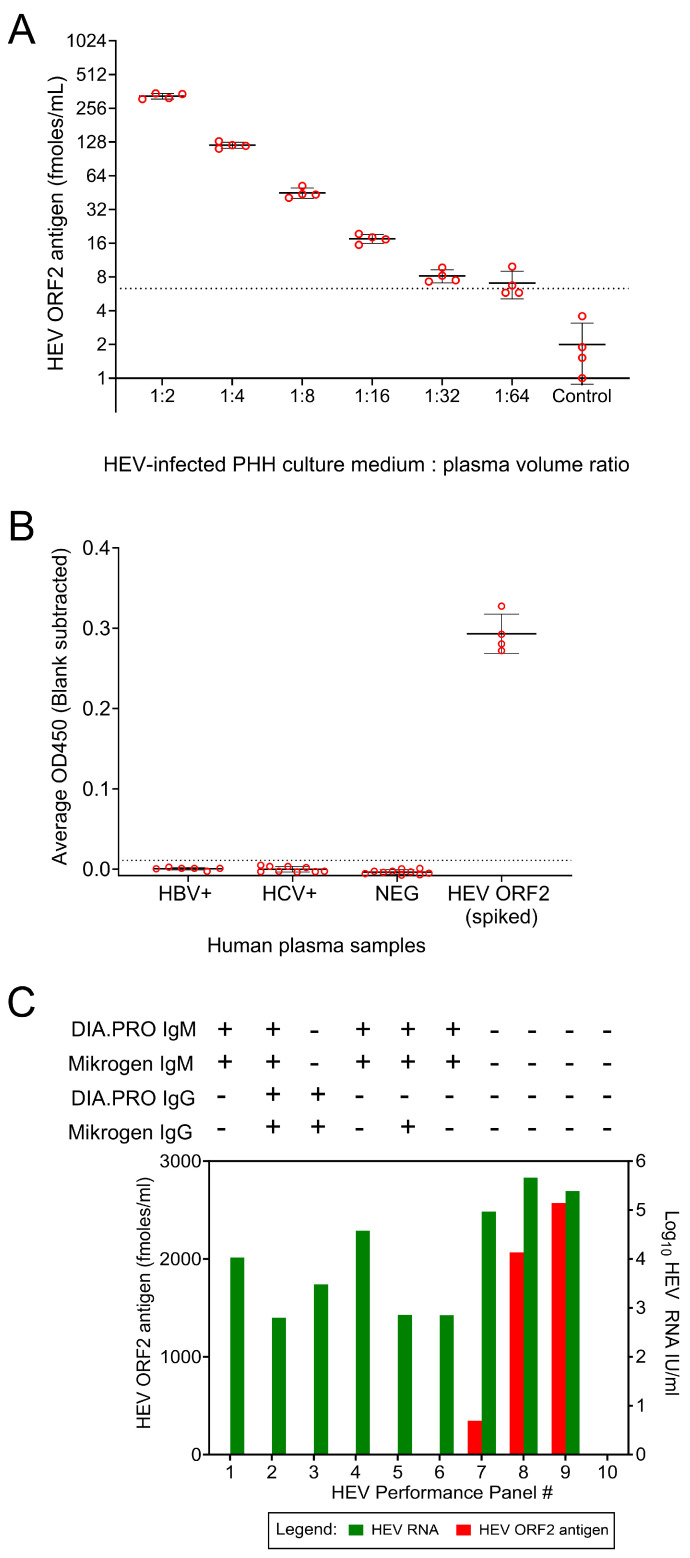
Quantification of HEV ORF2 protein in human plasma samples spiked with ORF2 antigen and the assay specificity assessment. (**A**) HEV ORF2 quantification in spiked human plasma samples. Human plasma samples were freshly prepared using blood samples from 4 different blood donors and individually spiked with HEV ORF2 protein from HEV-infected human hepatocyte culture medium at different volume ratios. The spiked human plasma samples were freeze-thawed once before performing ORF2 sandwich ELISA. The data are expressed as femtomoles (fmoles) per milliliter of plasma samples. Error bars indicate mean ± standard deviation. The LOD was determined as 6.3 (mean ± 3 SD), calculated using 10 negative plasma samples and indicated as dotted line. (**B**) The sandwich ELISA specificity was assessed with negative donor plasma samples (*n* = 10) as well as with Hepatitis B virus−positive (*n* = 6) or Hepatitis C virus−positive (*n* = 9) donor plasma samples. The cutoff was determined as 0.011 (mean ± 3 SD), calculated using 10 negative plasma samples and indicated as dotted line. Y-axis indicates mean OD450 value subtracted with blank OD450 value. Error bars indicate standard deviation. (**C**) The sandwich ELISA performance was assessed with a commercial HEV panel comprising human plasma samples positive for both HEV RNA and anti-HEV antibodies (panel # 1–6), positive for HEV RNA but negative for anti-HEV antibodies (panel # 7–9) and negative for both HEV RNA and antibodies (panel # 10). The data for anti-HEV IgM and IgG, as well as the HEV RNA copy numbers, are taken from the AccuSet HEV Performance Panel Datasheet provided by the manufacturer (Seracare; 0820-0503). DIA.PRO and Mikrogen are manufacturers of commercial anti-HEV IgM and IgG assays. IU—International Units. “+” and “−” signs represent reactivity and nonreactivity for anti-HEV IgM or IgG in the commercial assays.

The sandwich ELISA was further optimized to detect ORF2 in human plasma samples spiked with the medium from HEV-infected cultures of human hepatocytes at different dilutions. The assay LOD for the sandwich ELISA was calculated with negative human plasma samples (*n* = 10) and found to be 6.3 fmoles/mL (Mean ± 3 SD). ORF2 antigen was consistently detected in spiked human plasma samples at concentrations above 7.3 fmoles/mL (1:32 dilution) (Figure 6A). The specificity of the sandwich ELISA for HEV ORF2 antigen was assessed using plasma samples from blood donors that tested positive for HBV (*n* = 6) or HCV (*n* = 9), as well as plasma samples from blood donors that tested negative for transfusion-transmitted infections (*n* = 10). Human plasma samples spiked with culture medium from HEV-infected hepatocytes (1:4) were used as a positive control in the specificity assessment (*n* = 4). All HBV+, all HCV+, and all negative donor plasma samples showed OD450 values below the determined OD cutoff (0.011) (Figure 6B). Overall, the sandwich ELISA detected the ORF2 antigen of at least 7.3 fmoles/mL of human plasma samples spiked with the antigen, and this detection is specific.

To evaluate the performance of the ELISA in authentic human samples, a commercially available panel was tested that included plasma samples from multiple individuals who tested positive for HEV RNA. The panel included samples that were reactive for both viral RNA and anti-HEV antibodies and samples that were reactive for viral RNA but non-reactive for anti-HEV antibodies. One panel member (#10) was negative for both HEV RNA and antibodies against HEV and served as a negative control. ORF2 was detected in three of nine HEV RNA-positive samples (LOD—6.3 fmoles/mL). All three of the ORF2-reactive samples were non-reactive for both anti-HEV IgM and IgG. ORF2 antigen was not detectable in samples that were reactive for anti-HEV IgM or IgG (Figure 6C).

## 4. Discussion

We developed and optimized a quantitative sandwich ELISA for HEV ORF2 antigen using two novel monoclonal antibodies and biotin–streptavidin technology. Previous reports showed that HEV ORF2 antigen was detected in the blood of HEV-infected patients and blood donors [19,26] and experimental animals infected with HEV [20,27]. In experimentally infected monkeys, the ORF2 antigen was readily detected during the early phase of acute HEV infection before IgM antibodies appeared [27]. Similarly, ORF2 antigen was detected at high levels in chronic HEV patients when IgM/IgG antibodies were minimal or absent [16]. Immunological assays detecting viral antigens were used for the diagnosis of viral infections in clinical settings with limited resources [28]. Currently, there are no quantitative assays available for HEV ORF2 antigen. There is one qualitative sandwich ELISA (Wantai Biological Pharmacy, Beijing) available for HEV ORF2 antigen detection in clinical samples, which is being repurposed for the quantitation of ORF2 protein in an HEV-infected culture medium and experimental rabbit model using p239 virus-like particles as a standard [20,29]. Our sandwich ELISA is optimized for the quantitation of HEV ORF2 protein in different matrix types, including gerbil serum, human plasma, and the cell culture medium of HEV-infected human hepatocytes or hepatoma cells, utilizing the recombinant ORF2 p216 antigen (E2s dimer) as the standard. The assay showed significant cross-reactivity to ORF2 from the genotype 1 and 4 strains. It successfully detected low levels of ORF2 antigen secreted in the culture medium of human hepatocytes infected with the HEV GT3 strain or Huh7 cells transfected with HEV GT3 RNA. The assay was used to assess and compare the magnitude and duration of ORF2 antigen detection in the serum of gerbils experimentally infected with HEV GT3 with or without immunosuppressive drug treatment. Our sandwich ELISA detected at least 359 pg/mL (equivalent to 7.3 fmoles/mL) of HEV ORF2 antigen in human plasma samples spiked with HEV GT3 ORF2 from a culture medium of infected human hepatocytes. The assay successfully detected the HEV ORF2 antigen in human plasma samples positive for HEV RNA but negative for anti-HEV antibodies. The assay was specific for HEV antigen because donor plasma samples positive for other hepatitis viruses (HBV and HCV) were non-reactive in the assay.

A previous report suggested polyclonal antibodies are not suitable for capturing the HEV ORF2 antigen, while monoclonal antibodies are efficient in capture and detection of ORF2 antigen in a sandwich immunoassay format [27]. Moreover, indirect biotin amplification technology improved the antigen detection efficiency necessary for the analysis of clinical samples [27]. We developed a panel of mouse monoclonal antibodies specific to an HEV GT3 (US-2) ORF2, targeting E2s dimer [30] and identified 8A8 mAb as the best antibody to capture ORF2 antigen in the solid phase and 7D3 as the best detection antibody when using the biotin–streptavidin complex. When 8A8 mAb was both the capture and detection Ab in the competition experiments, a negative result was obtained in all Ab concentrations tested (up to 1000 ng/mL). This phenomenon is more commonly observed in high-affinity antibodies specific to the ORF2 E2s dimer [31,32]. On the other hand, the 7D3 antibody yielded a positive result when it was both the capture and detection Ab, indicating that two 7D3 molecules can separately bind to two epitopes on the ORF2 dimer in a noncompetitive manner. Few E2s-specific antibodies nonblocking to themselves were previously reported [32].

The HEV ORF2 protein has three isoforms differing in molecular weight, protein structure, and glycosylation pattern [20,21]. Both capture and detection antibodies were developed specific to the protruding domain (P domain) [33], which is present in all three isoforms of the ORF2 protein. Nevertheless, the secreted isoform of ORF2 (ORF2S) and its truncated versions are the exclusive targets (more than 99.9%) of the ORF2 antigen ELISA in the hepatocyte culture medium, in the patient sera and urine samples [20,34,35]. Consistent with this, our sandwich ELISA detected large amounts of native ORF2S in the culture medium of human hepatocytes infected with HEV genotype 3 (Kernow C1 p6) and of Huh7 cells transfected with capped HEV RNA. Uncapped HEV RNA can initiate HEV replication, although inefficiently, and secrete low amounts of ORF2 antigen into culture supernatants [36]. Consistent with this, our assay detected low levels of ORF2 antigen secreted from Huh7 cells transfected with uncapped HEV Kernow C1 p6 RNA.

A previous study identified eight distinct groups of ORF2 variants in HEV patients showing continued viral infection despite ribavirin therapy [37]. These ORF2 variants possess single amino acid substitutions in the region between amino acid positions 25 to 324. The monoclonal antibodies 8A8 and 7D3 bind to domains outside this region (position 422–637); therefore, our sandwich ELISA is expected to detect these natural ORF2 variants in patients’ plasma. The antigenic target of our assay has a highly conserved glycosylation site at N562, and only six out of 261 sequences in GenBank showed a mutation (D562) at this site [38], which may affect the antibody binding to the antigen in our assay. Indeed, our assay successfully detected non-glycosylated forms of ORF2 p216 antigen produced in bacteria, suggesting N-glycan at position 562 is dispensable for binding of both 8A8 (coating) and 7D3 (detection) antibodies to the antigen in our assay.

The HEV ORF2 antigen was detected in the blood of both acute and chronic HEV patients, and the HEV chronicity is associated with long-term immunosuppressive drug treatment [16]. In the absence of significant antibody responses, HEV ORF2 was readily detectable in blood during the initial phase of acute HEV infection in experimentally infected monkeys [27]. Consistent with this, gerbils showed significant levels of ORF2 antigen in serum in the first 2 weeks after HEV infection when the antibody response was absent or minimal [25]. However, the ORF2 antigen was not detectable from 3 wpi when most serum samples turned positive for HEV IgM and IgG [25]. This is consistent with the report that ORF2 antigen was not detected in the majority of serum samples of acute HEV patients positive for HEV IgM and IgG [27]. Moreover, ORF2 protein was found at persistently high levels in the serum of immunosuppressed gerbils, as also observed in solid organ transplant recipients during acute HEV infection that eventually progressed to chronicity [39]. We found that ORF2 antigen levels were strongly correlated with HEV RNA levels in gerbil serum, similar to that reported in acute HEV-infected patients [12,17].

The sensitivity of ORF2 antigen detection is lower compared to HEV RNA detection in clinical samples [16,40]. Recent studies showed that urine samples are more suitable for sensitive detection of ORF2 antigen as compared to serum [35] but not suitable for discriminating acute and chronic HEV patients [39]. Our sandwich ELISA was developed specifically for the ORF2 E2s antigen and is, therefore, predicted to detect the ORF2 E2s-like antigen (amino acids—459–606) excreted in urine samples [35]. Further studies are warranted to evaluate the utility of our sandwich ELISA in detecting the HEV ORF2 antigen in urine samples.

To assess the clinical sensitivity and specificity of the sandwich ELISA, the assay needs to be evaluated with known HEV-positive and -negative clinical samples [15]. Because HEV-positive clinical samples are rare and difficult to obtain in North America, we analyzed the analytical sensitivity of the assay with human plasma samples spiked with different concentrations of HEV GT3 ORF2 from infected human hepatocyte culture medium. The HEV ORF2 antigen was detected efficiently close to the limit of detection of the assay in spiked human plasma samples, suggesting the assay may be suitable to test human plasma samples from HEV GT3-infected patients containing low amounts of the ORF2 antigen. When tested with a commercial HEV panel comprising human plasma samples positive for HEV RNA, our ELISA detected the ORF2 antigen only in samples negative for anti-HEV antibodies, similar to the observation in acute HEV patients [27]. Further, the assay was found to be specific to the HEV ORF2 antigen as plasma samples positive for other hepatitis viruses, such as HBV and HCV, as well as negative control plasma samples were non-reactive in our ELISA.

In conclusion, we developed and optimized a novel sandwich ELISA to detect and quantify the HEV ORF2 protein in different sample matrix types utilizing an ORF2 standard. Our study demonstrated the ORF2 antigen ELISA may be used to monitor HEV infection in vitro in a human hepatocyte culture medium, assess the magnitude and duration of acute and chronic HEV infection in a gerbil model, and detect active HEV infection in human plasma samples from HEV-infected patients negative for anti-HEV IgM or IgG. The sandwich ELISA detected ORF2 antigen produced in two different species, human cells and gerbils, using a secondary detection system (Streptavidin–HRP) that is suitable for samples from other species as well. Therefore, our sandwich ELISA is expected to detect the HEV antigen in serum and plasma samples from other animal models of the HEV infection, such as rhesus macaque, pigs, rabbits, and rats. Additional studies are needed to assess the clinical utility of the ORF2 antigen ELISA in the screening and diagnosis of HEV infections in humans.

## Data Availability

All data are contained within the article.

## Supplementary Materials

The following supporting information can be downloaded at: https://www.mdpi.com/article/10.3390/v16030393/s1, Figure S1: The experimental design of sandwich ELISA development and optimization for HEV ORF2 antigen; Figure S2: Checkerboard titration to determine concentrations of coating 8A8 mAb (c8A8) and detection 7D3 mAb (d7D3) in sandwich ELISA format using recombinant ORF2 p216 antigen.
