# Supplementary material for: Quantification of Hepatitis E Virus ORF2 Protein by a Novel Sandwich ELISA
Source: Viruses. 2024 Mar 2;16(3):393. doi: 10.3390/v16030393 (PMC10974087; doi:10.3390/v16030393)
Supplement: Supplementary file 1 [file viruses-16-00393-s001.zip › viruses-2806176-supplementary.pdf]

SUPPLEMENTARY INFORMATION:

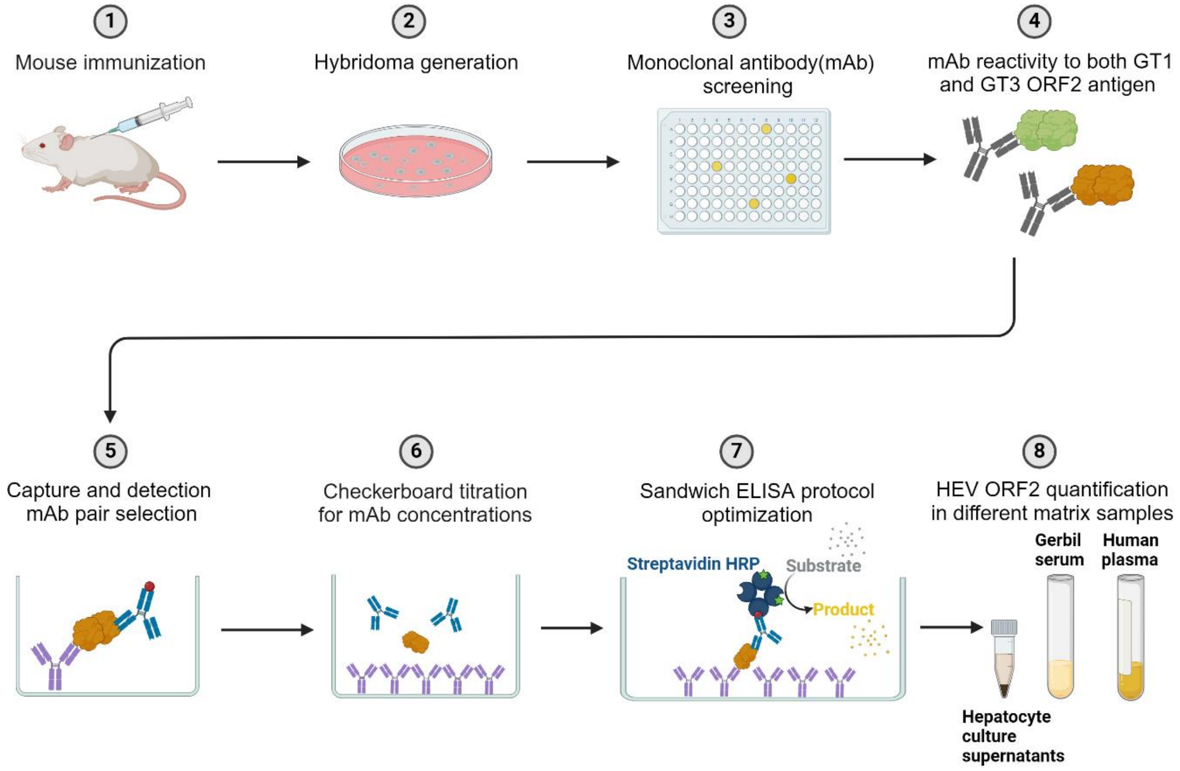

Supplementary Figure 1. The experimental design of sandwich ELISA development and optimization for HEV ORF2 antigen. (Illustration is made using tools at Biorender.com)

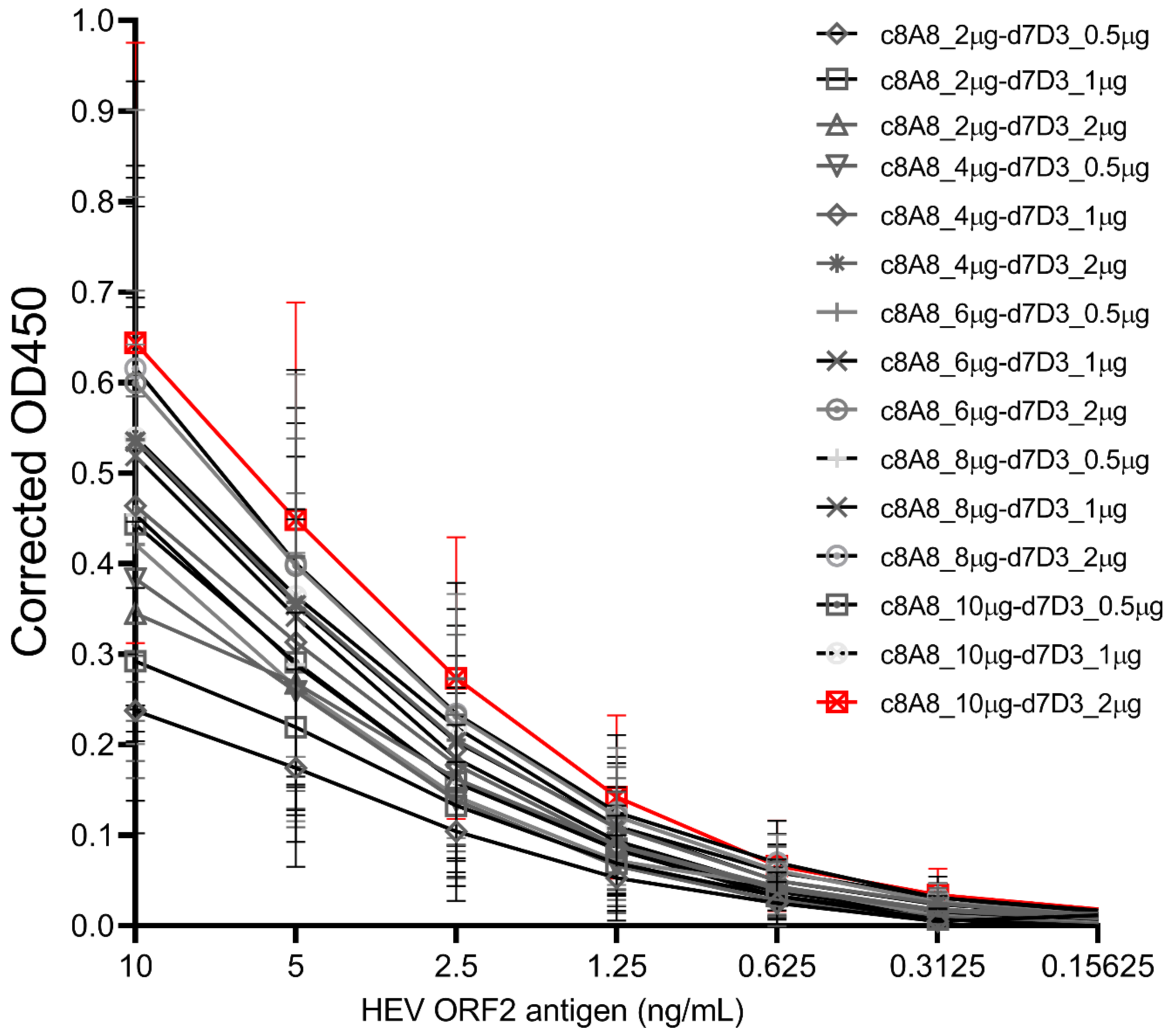

Supplementary Figure 2. Checkerboard titration to determine concentrations of coating 8A8 mAb (c8A8) and detection 7D3 mAb (d7D3) in sandwich ELISA format using recombinant ORF2 p216 antigen. c8A8 was tested at five concentrations (2, 4, 6, 8, and 10µg/ml) against d7D3 that was tested at three concentrations (0.5, 1, and 2µg/ml). Each data set (indicated with a different symbol) is derived from testing one combination of c8A8 and d7D3 concentrations in the sandwich ELISA format using a series of 2-fold diluted recombinant ORF2 p216 antigen. The secondary reagent (Streptavidin-HRP) was used at a dilution of 1: 20000. The corrected OD450 value in the y-axis is derived from subtracting OD450 value of each c8A8-d7D3 concentration pair tested from

that of negative control (blocking buffer only, no antigen) specific to that concentration pair. The best antibody concentration pair is indicated in red showing highest corrected OD450 in multiple antigen concentrations tested. The negative controls of all concentration pairs tested showed OD450 less than 0.1. Data is from 3 independent experiments done with 3 different batches of c8A8 and d7D3, and 3 different commercial lots of Streptavidin-HRP. ELISA was done with 2 technical replicates and the OD450 values of replicates were averaged for each concentration pair before calculating corrected OD450. Error bars represent standard deviation.
